# Rising HIV seroconversion rates & associated risks among employees of organization ‘X’: A case control study, Pakistan, 2017

**DOI:** 10.12669/pjms.36.6.1735

**Published:** 2020

**Authors:** Eisha Mansoor, Naila Azam, Saifullah Khan Niazi, Naveen Sheikh, Mirza Amir Baig, Mansoor Tariq Azim, Nimra Klair

**Affiliations:** 1 Eisha Mansoor, MBBS. Armed Forces Post Graduate Medical Institute, Rawalpindi, Pakistan; 2 Naila Azam, FCPS (Community Medicine), MCPS (Family Medicine). Armed Forces Post Graduate Medical Institute, Rawalpindi, Pakistan; 3 Saifullah Khan Niazi, FCPS (Virology). Armed Forces Institute of Pathology, Rawalpindi, Pakistan; 4 Naveen Sheikh, Medical Student, Army Medical College, Rawalpindi, Pakistan; 5 Mirza Amir Baig, MPH, MHM. National Institute of Health, Islamabad - Pakistan; 6 Mansoor Tariq Azim, MBBS, FCPS (General Surgery). Military Hospital, Rawalpindi, Pakistan; 7 Nimra Klair, Medical Student, Medical Student, Army Medical College, Rawalpindi, Pakistan

**Keywords:** HIV Seropositivity, HIV, seroconversion

## Abstract

**Background and Objectives:**

In 2004 Pakistan escalated from ‘low-prevalence’ to ‘concentrated’ phase of HIV epidemic. Despite global decline in HIV incidence since 1997, rate of HIV infections in Pakistan is persistently rising since 1990. Available literature focusses on key populations or localized outbreaks limited by short study duration and regional applicability of results. We studied HIV seroconversion trends over a period of 8 years in a geographically diverse population and evaluated associated risk factors.

**Methods:**

A desk review of HIV surveillance data from 2010 to 2017 was carried out at Armed Forces Institute of Pathology. A case was defined as any adult employed in organization ‘X’, initially screened for HIV but later seroconverted on ELISA and western blot. Case-control study was conducted on cases diagnosed in 2017. Age and sex matched controls were identified from same population sub-group. Structured telephonic interviews were conducted and statistical analysis done at 5% margin of error.

**Results:**

The annual HIV diagnosis rate remained relatively stable till 2015 (< 40 /100,000/yr) after which it rose sharply to 60/100,000/yr in 2016 .Upward trend continued in 2017 to reach 125/100,000/yr (>200% increase from baseline). Acquisition of HIV was significantly associated with commercial sex activities (OR=9; 95% CI: 1.25-395).

**Conclusion:**

HIV seroconversion rates among employees of organization X have increased significantly in the past two years. Unlike HIV outbreaks previously reported from Pakistan, sexual route seems to be the predominant mode of transmission. Focus is mandated on prevention of sexual transmission of HIV at national level as well for all vulnerable populations.

## INTRODUCTION

In 2004, Pakistan escalated from ‘low prevalence’ to ‘concentrated’ phase of HIV epidemic.^1^,^2^ Sporadic cases as well as outbreaks of HIV are increasingly being reported from many parts of the country.^3^ Progression of HIV in Pakistan is believed to be following the Asian Epidemic Model (AEM).^4^ However, Pakistan has followed a different trend from its contemporaries in the epidemiologic transition of other infectious diseases. Besides being among the last few countries to eradicate Small Pox, it continues to lag behind the globe in Polio eradication as well.^5^,^6^ The tale of HIV AIDS in the country also presents a similar picture. World over, the tide of HIV epidemic was checked in 1997 when global incidence of HIV infections started to fall.^7^ However, Pakistan is among the few countries where HIV cases are persistently increasing since 1990.^4^,^8^ Evidence based forecasts suggest that the rising trend is likely to continue.^9^

The key to reversing this tide lies in effective surveillance of HIV in all vulnerable populations.^10^ Available studies done on HIV in Pakistan are mostly focused on Persons Who Inject Drugs (PWID) / sex workers or are investigations of localized outbreaks.^11^ There is a paucity of data on HIV dynamics in other high risk populations like migrant workers as well as in general population. Local outbreak investigations are limited by short duration of follow up and regional applicability of results. We sought to study HIV trends over a period of 8 years among employees of organization ‘X’; which was a geographically diverse population. The study population was comparable to various bridging populations in the country which can play a crucial role in paving way for a generalized HIV epidemic taking course in Pakistan.^12^ The objectives were to analyze the trend of HIV infections in a bridging population, identify the risk factors responsible for the growing HIV epidemic and to come up with reliable recommendations for checking the tide of HIV.

## METHODS

Secondary analysis of HIV surveillance data was conducted at Armed Forces Institute of Pathology (AFIP) coupled with a case control study at Armed Forces Post Graduate Medical Institute (AFPGMI). All the cases / records of HIV positive employees available from January 2010 to December 2017 were included in the study (universal sampling). Study population comprised around 80,000 men working in a country wide multidimensional organization ‘X’. They were deployed all over the country with main office in federal capital Islamabad. They were contracted for a variety of roles like administrators, engineers, laborers, cooks, telephone operators, security staff, health staff, and many more. They hailed from all regions of the country and were demographically comparable to the general population of Pakistan. The organization is on the panel of Army Medical Corps for full medical insurance of all employees. Therefore the employees are entitled to free medical care in military hospitals; hence remain under medical surveillance in the same organized way as uniformed army personnel. Yet they do not come under the Pakistan Military Law; so their discipline and behaviors remain similar to Pakistan’s general population. Therefore, we studied the rich and reliable HIV surveillance data available for these individuals to understand the HIV dynamics in Pakistan’s general population and similar bridging populations.

A case was defined according to the WHO Case Definition of HIV as an adult person employed in organization ‘X’, initially screened for HIV and later found to have positive HIV antibody testing (rapid or laboratory-based enzyme immunoassay), confirmed by a second HIV antibody test relying on different antigens or of different operating characteristics; and/or; positive virological test for HIV or its components (HIV-RNA or HIV-DNA or ultrasensitive HIV p24 antigen) confirmed by a second virological test obtained from a separate determination. All employees meeting the case definition from 2010 to 2017 were identified through the computerized laboratory database at Armed Forces Institute of Pathology (AFIP). A case-control study was planned using 22 cases diagnosed in 2017. Eighty eight age and sex matched controls were selected in a case to control ratio of 1:4. Controls were chosen from among those workers who had a negative HIV lab test during HIV screening done for non-clinical reasons e.g. as a prerequisite for blood donation / surgery etc.

To extract the relevant data from lab records, we trained three medical students for collecting and compiling pertinent information from surveillance records. A desk review of extracted data was carried out to allow time trend analysis. For case control study, a structured interview Form was developed and pretested to elicit relevant demographic and risk factor information from selected cases and controls. We developed the interview tool using Canadian AIDS Society Guidelines for Assessing Risk of HIV Transmission. Considering gender sensitivities and the personal nature of questions included in the questionnaire, interviews were conducted on telephone by a male Preventive Health Assistant (PHA) at AFPGMI after appropriate training. Telephonic interviews were also more feasible as workers had been repatriated to their homes on being diagnosed with HIV as per organizational policy. Verbal informed consent was taken prior to each interview. A positive history of blood transfusion, surgical procedure, extramarital sexual contact, use of community barber shops, substance abuse, tattooing / body piercing and travel to high HIV prevalence region e.g. Sub-Saharan Africa during 10 years prior to diagnosis was considered as a risk factor for HIV acquisition.

Filled interview forms were handed over to a medical student to extract relevant data from interview forms and enter into Microsoft excel. Descriptive and advanced statistical analyses were performed using Epi-info version seven to test associations between potential risk factors and diagnosed HIV infections. Exposure rates and Mental Henzel Odds Ratios (OR) were calculated for all risk factors. Fisher exact test was applied keeping 95% confidence interval and a p value<0.05 as significant.

## RESULTS

From 2010-2015, annual number of HIV cases among study population remained around 12 which started to rise sharply from 2015 onwards to reach 29 in 2017 as shown in the line graph below.

HIV diagnosis rates were also calculated for every year using population denominators from payrolls to rule out the possibility of an apparent rise in HIV positive tests due to increase in size of study population. Calculated rates were plotted as under and a similar trend was observed.

**Fig.1:**
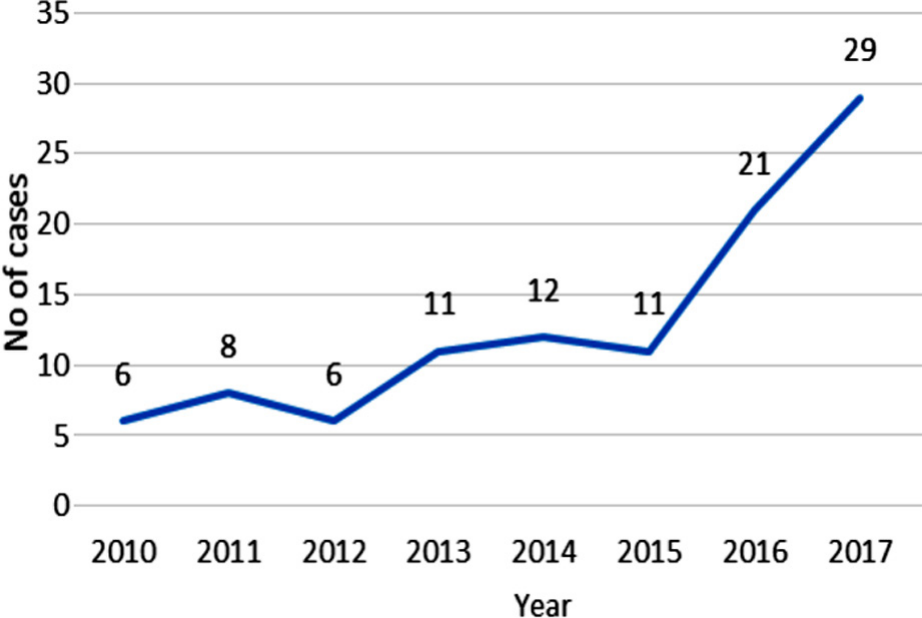
Annual HIV Cases among Employees of Organization X: Pakistan, 2010-17.

**Fig.2:**
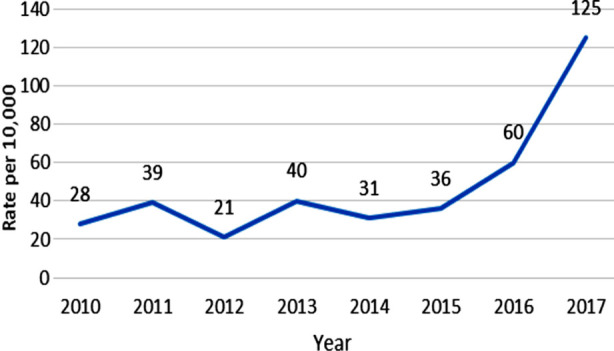
Annual HIV diagnosis rate among employees of organization ‘X’: Pakistan, 2010-2017.

From 2010-2015, HIV diagnosis rate remained <40/10,000/year which rose to 60/10,000/year in 2016.Upward trend continued in 2017 to reach 125/10,000/year (>200% increase from baseline).Hence rates remained relatively stable till 2015 after which a sharp increase was seen.

Twenty nine cases of HIV were diagnosed in 2107 out of which, 22 (76%) cases were included in the case control study. Remaining seven cases were excluded, because their contact information could not be traced (n=2), declined to take interview (n=1), died (n=1), or did not respond within 10 call attempts (n=3). All were male with a median age of 35.18 years (Range= 25-52). Married to single ratio was 5: 1. Average income was Rupees 16,350 /per month (Range 13,000-18,000). Seventeen cases (77%) were symptomatic while 5 (23%) were found to be HIV positive on routine screening prior to blood donation (n=4) or on contact tracing (n=1). Common clinical presentations included fever (n=9, 41.67%), skin ulcer (n=5, 25%), diarrhea (n=4, 16.7%) and weight loss (n=4, 16.7%). Four cases (16.7%) gave history of other medical disorders as well including Hepatitis (n=1), Diabetes mellitus (n=1), Idiopathic thrombocytopenia Purpura (n=1) and Tuberculosis (n=1). Out of 22 HIV positive workers, 10 (45%) were from Sindh province, 7 (32%) from Punjab, and 2 (9%) each from KPK and AJK and 1 (5%) from IC

To find the association of identified risk factors with acquisition of HIV, Odds Ratios (OR), Mantel-Hansel Odds Ratios (OR_MH_), P values and Confidence Intervals (CI) were calculated as shown in Table-I (n=22). Hence commercial sex was the only risk factor found to be significantly associated with acquiring HIV infection in the group under study. Other prevalent risk factors were visits to barber shops and history of surgery.

**Table-I:** Univariate analysis of risk factors among employees of organization X: Pakistan, 2017.

Risk factor	Exposure rates cases No (%)	Exposure rates controls No (%)	OR _MH_	P value	95%CI
Commercial Sex	8(36)	10(11)	9.0	0.01	1.25-395
Barber shop shave	18(82)	60(68)	7.1	1.00	0.36-135
Surgery	7(32)	24(27)	4.8	0.10	0.55-237
Dental procedure	4(17)	4(5)	3.4	0.31	0.24-157
Blood transfusion	4(17)	12(14)	1.5	0.50	0.17-18
High risk travel	1(5)	3(3)	1.1	0.43	0.21-23
Body piercing	3(14)	0	-	-	-
Substance abuse	3(14)	0	-	-	-

## DISCUSSION

Rising incidence of HIV observed in our study is consistent with the rising trend of HIV in Pakistan’s general population as well as known key populations. However, the rate of HIV diagnosis observed in our study population is higher than that observed in other populations. The rate of HIV seroconversion among employees increased by 50% from 2015 -2016 and further by 108% from 2016-2017. In comparison, HIV prevalence among PWID (Persons Who Inject Drugs) remained relatively steady with a value of 37.8% in 2011 to 38% in 2016 increasing by less than 1 % per year.^13^,^14^ The relatively higher rates of increase in our study could be attributed to surveillance bias assuming that disease surveillance may be more sensitive in populations dependent on army owing army’s efficient and organized health system. However, it is unlikely considering our study population included civilian employees who were did not undergo any additional HIV screening and their HIV status was revealed incidentally. Moreover the difference in rates is much larger than can be accounted for by surveillance bias alone. So it can be concluded that HIV prevalence in bridging populations and general population may be low in absolute figures but might be increasing at a faster pace. These findings call for a need to direct preventive and control efforts towards general population as well alongside the key high risk populations like PWID. The relatively stable rate of HIV infections in PWIDs over the past few years observed in Pakistan prove that preventive and control efforts do have the potential to check the soaring rates in other populations too.

All other known confounders which may also produce an apparent increase in reporting of infections were also taken into consideration. The possibility of Lab error was negligible considering that all HIV positive cases at AFIP were tested by ELISA method and reconfirmed by a 2^nd^ ELISA or chemiluminiscence method (e.g. Abbot Architect / Vitros, Ortho Clinical). Weak positive cases or cases with discordant results were further tested by a confirmatory assay (Bio – Rad Geenius HIV ½), also known as Western blot. It was also verified from AFIP that the reported increase in HIV cases was not due to any change in local reporting / diagnostic procedure or case definition, posting of a new physician / infection control nurse or upgradation of a healthcare facility. Therefore it can be said with certainty that the rising rate of HIV infections shown in Table-I represents a true rise in incidence of HIV in our study population.

Predominance of males in our study group is understandable considering that all subjects were workers who had settled in various cities for better job prospects. Most HIV positive workers in our study belonged to Sindh (n=10, 45%) and Punjab (n=7, 32%). Place distribution of HIV cases also corresponds to the national data. Six out of the eight major HIV outbreaks in the country have been reported from Sindh and Punjab. The recent upsurge of HIV in Larkana and Faisalabad also portray an alarming picture in these two provinces.^3^ Areas with high transmission potential can be targeted for high impact interventions.^15^ However, without knowing the province wise distribution of all the employees of the organization to calculate province wise rates for comparison, we cannot comment on the extent to which the observed differences in place-distribution of cases depict the true picture.

With regards to risk factors, sexual route was recognized as the predominant mode of transmission of HIV in study population.^16^ Although globally, transmission through sexual contact is estimated to account for 75 to 85 percent of cumulative cases of HIV / AIDS); such an association has been difficult to document in a Muslim majority country like Pakistan.^17^ Some degree of reluctance also exists from the government to address the issue head on.^18^ This lack of acceptance may be one of the reasons for inadequate HIV control in Pakistan.^19^ Recognizing the association of HIV epidemic with unprotected sex could help steer our policies towards improved HIV prevention and control.^20^

Presently, the AIDS control suffers from a stagnation of prevention efforts.^21^ Limited available resources are dedicated to controlling spread of HIV via blood transfusion, surgery, organ transplantation etc. alongside provision of expensive retroviral drugs, but the incidence continues to rise; indicating probable oversight of an important factor responsible for spread of HIV in Pakistan. Considering this as a case of “social denial”, strategies to check HIV transmission through high risk sexual behavior may hence have the potential to make a difference.

Another salient feature of this study is the population under consideration as there is a paucity of literature explaining HIV trends among such bridging populations. Available literature focusses on HIV trends in four key populations namely PWID (Persons Who Inject Drugs), MSM (Men who have Sex with Men), MSW (Male Sex Workers) and FSW (Female Sex Workers).^22^ Pakistan has already entered in the concentrated phase of HIV epidemic having >5% prevalence persistently in two of these key populations. What awaits Pakistan now is the potential risk of a generalized epidemic. Therefore, at this stage, it is imperative that we start looking beyond the four key populations and address to other bridging populations in the country. Such populations are more closely related to the general population; yet escape attention. So they may serve as a pertinent bridging group to take HIV infection from key populations to general population; hence innocuously expanding HIV epidemic in Pakistan from the present “concentrated stage” to the third and final stage of a “generalized epidemic”.^23^ These individuals are also important being the sole bread earners of their families. Loss of this productive population to a life threatening disease would therefore complicate the socioeconomic fabric of the society.

To check the rising incidence of HIV, due regard must be given to all vulnerable segments of society as well as general population alongside the known key populations at risk. There is a need to reemphasize on prevention of new HIV infections including strategies to curb high risk sexual behavior especially among those living under restricted environments away from families.

### Authors’ Contribution:

**EM:** Principle investigator, conducted the study, did advanced statistical analysis & writing of manuscript.

**NA:** Conceived, designed & supervised the study, and final approval of manuscript.

**SKN:** Did laboratory related analysis, acquisition of data and manuscript writing

**NS, NK:** Did selection of controls and data collection / entry/ basic analysis.

**MAB:** Planned methodology, statistical analysis & writing of manuscript.

**MTA:** Data collection, data cleaning, interpretation of results and clinical correlation.
